# Depression in Multiple Sclerosis: A Review of Assessment and Treatment Approaches in Adult and Pediatric Populations

**DOI:** 10.5402/2012/427102

**Published:** 2012-10-14

**Authors:** Maria Skokou, Evanthia Soubasi, Philippos Gourzis

**Affiliations:** Department of Psychiatry, School of Medicine and University Hospital of Patras, University of Patras, Rio, 26504 Patras, Greece

## Abstract

Multiple sclerosis is a chronic demyelinating disease affecting one million people worldwide, with a significant burden of psychiatric comorbidity. Depression is the commonest psychiatric manifestation but still remains largely underdiagnosed and undertreated. The present work reviews current knowledge on diagnosis, assessment, and somatic and psychotherapeutic treatment interventions for depression in adult and pediatric populations of patients with multiple sclerosis.

## 1. Introduction

Multiple sclerosis is a chronic demyelinating disease of the central nervous system, affecting any part of the CNS, but mostly white matter tracts in the cerebral hemispheres, optic nerves, brainstem, cerebellum, and spinal cord [1]. The prevalence of the disorder ranges between 2 and 150/100,000 depending on the country or specific population [2], affecting approximately 1 million people worldwide [3]. Pediatric-onset cases comprise 2–5% of total multiple sclerosis patients [4].

Psychiatric symptoms in the context of MS were first noted by Charcot, who described mania, hallucinations, and depression among other manifestations of the disease [5]. Lifetime prevalence of major depression ranges from 19 to 54%, depending on the population sample and diagnostic criteria used [6–8]. Annual prevalence is estimated at 16% [9], and point prevalence of clinically significant depressive symptoms is as high as 50% [10]. The rate of depressive disorders in a pediatric population with demyelinating disease has been estimated at 27% [11]. Evidently, prevalence is considerably higher than in the general population or among patients with general medical conditions other than MS [8]. Etiologic factors seem to be both biological and psychosocial [12], and risk factors have been reported to be female sex, age <35 years, family history of major depression, and a high level of stress [13]. Disease-modifying agents, interferon beta in particular, have also been suspected to induce depressive symptomatology [14, 15]. However, subsequent well-designed studies failed to prove a depressogenic effect, and pretreatment depressed mood was found to be the best predictor of subsequent depression [16, 17]. The importance of this clinical issue cannot be underestimated, given its high frequency, the possible adverse effects of depression on disease course [18], cognitive functioning [19], treatment adherence [20], deleterious consequences on quality of life [21], and increased risk of suicide [22, 23]. 

Unfortunately, mental comorbidity and depression in particular are frequently underdiagnosed and undertreated, with reported rates of missed diagnosis around 23–30% [6, 24] and rates of inadequate treatment around 20%–36% of those reporting depression [6, 25]. In a cross-sectional community study of patients with MS by Cetin et al. [26], 59% of the patients who had significant symptoms of depression were not taking medication, possibly due to missed diagnosis, denial of symptoms, or false reporting, whereas the rest 41% received inadequate treatment. In the whole sample (*n* = 542), 28% of the patients were adequately treated for depression and therefore had achieved remission. In another study by Mohr et al. [27], 65.6% of depressed patients did not receive medication, 4,7% received subthreshold treatment, 26,6% received threshold, and only 3.1% received overthreshold, indicating that in most cases, therapy is not in accordance with treatment guidelines for depression. Given the fact that depression is a treatable condition, correct diagnosis and adequate treatment are issues of immense importance for the management of multiple sclerosis patients.

The present paper is focused on current knowledge on diagnosis, assessment, and therapeutic interventions for depression in the context of multiple sclerosis. 

## 2. Diagnosis and Assessment

### 2.1. General Considerations

Although the symptom of depressive mood is almost always experienced by patients suffering from disabling diseases, the syndrome of major depression, as defined by the classification systems of ICD-10 [28] and DSM-IV-TR [29], corresponds to a constellation of symptoms comprising depressive mood, anhedonia, fatigue, psychomotor retardation or restlessness, suicidal ideation or suicide attempt, feelings of guilt and worthlessness, and difficulty in concentrating, as well as vegetative symptoms, including altered sleep, appetite, and sexual arousal [30]. A common challenge for correctly diagnosing depression in the context of multiple sclerosis is distinguishing whether a certain symptom emanates from a depressive disorder or can be attributed to the demyelinating disease. Potential confounders are fatigue, insomnia, altered appetite, and impaired memory and concentration, and misjudgments will lead to false positives or false negatives. Varied presentations of depression, for example, unexplained somatic complaints, anxiety, or hopelessness instead of sadness, could also complicate diagnosis, especially in older persons [31]. Patients with MS might exhibit pathological laughter or crying occurring with bilateral forebrain disease, which poorly correlates with underlying mood [1]. On the other hand, depression adversely affects appraisal of physical symptoms [32, 33]. A way of disentangling the differential diagnosis in such cases is by paying attention to the cognitive and affective domains of the depressive symptomatology, for example, the depressive mood and depressive beliefs, which is also an approach adopted by assessment tools and rating scales for depression in the presence of a physical illness and multiple sclerosis in particular [12].

### 2.2. Screening and Assessment Tools

The use of two screening questions [34] has been proposed for detecting major depressive disorder in the context of MS and primary care setting [35], which correspond to depressive mood (“during the last two weeks, have you often been bothered by feeling down, depressed, or hopeless?”) and anhedonia (“during the past two weeks, have you often been bothered by little interest or pleasure in doing things?”). At least one affirmative response on either question was used as a criterion of having MDD. This approach was shown to have a positive predictive value of 71.7%, with a rate of 27.3% of false positives. However, two thirds of false positives were shown to have subthreshold depression, which again should alert the clinician for the need of treatment [35]. Using the Yale single question [36] is even less time consuming, but this tool seems to lack sensitivity, as it could not identify 34.7% of patients who were depressed by Beck Depression Inventory criteria. On the other hand, it appears to be quite specific, that is, depression can be ruled out when it does not exist [37].

 The Beck Depression Inventory [38] is an objective self-report assessment tool comprising 21 items, and one of the most commonly used for patients with multiple sclerosis. Recommended cutoff point is set at 13, though 30% of patients with depression will be missed in this way [8]. Concerns regarding the length of the instrument as well as the inclusion of items corresponding to neurovegetative symptoms, which might lead to overdiagnosis, have led to the implementation of shorter forms, such as the 7-item Beck Depression Inventory-Fast Screen (BDI-FS) [39]. Validity of the instrument has been documented for the population of patients with multiple sclerosis [40]. 

The Hospital Anxiety and Depression Scale [41] is a self-assessed questionnaire consisting of 14 items, suitable for use in persons with medical conditions, if items corresponding to somatic symptoms are omitted [42], and recently validated for people with multiple sclerosis [43]. 

The Center for Epidemiologic Studies Depression Rating Scale (CES-D) [44], comprising 20 items, has been used with a cutoff point of 16 (>15), as likely to correspond to significant depression, recognizing 74.5% of depressed persons [9, 45]. Another useful scale is the Chicago Multiscale Depression Inventory that was developed to assess depression in MS and other chronic diseases [46]. It is a 42-item self-reporting questionnaire consisting of three subscales, mood, physical malfunctioning, and self-criticism, the first of which is recommended for MS [47]. The validity of the use of the Hamilton Rating Scale for Depression [48] has been examined in one study [49]. The authors conclude that the whole or part of the scale can be used, depending on study design. The Depressive Mood Scale (EHD, Echelle d' Humeur Dépressive) is an 11-item French questionnaire specifically designed and validated for the assessment of depression in MS, focusing on mood changes rather than somatic symptoms [50]. The Zung Self-Rating Depression Scale (ZSRD) [51] is a 20-item tool that has been shown to have good construct validity for measuring depression in medically ill populations [52] and has been used for patients with MS [53, 54], as well as the Montgomery-Asberg Rating Scale (MADRS) [55], a widely known clinician-rated assessment tool for depression, consisting of 10 items [56]. 

In pediatric populations, researchers most frequently use the Child Depression Inventory for identifying depression in samples with MS [57, 58]. The presence of depression can also be documented by applying the Kiddie-SADS interview [59].

The utility of detecting depression partly depends on the efficacy and availability of treatments as well as willingness of patients to receive treatment. In a recent study, less than 30% of depressive individuals who were prompted to receive treatment did so, and possible explanations for this were distorted beliefs of depression being inevitable, practical problems, or the depression itself [60, 61]. Concluding, identifying depression, though not sufficient to ensure effective treatment, is a necessary first step towards proper management of depressed patients with multiple sclerosis. 

## 3. Treatment Approaches

### 3.1. General Considerations

A comprehensive treatment plan for depression should include pharmacotherapy, psychotherapy, or cognitive behavioural therapy in specific or combination therapy. Screening for suicidal intend is incremental due to the high cumulative lifetime risk for suicide [7].

Treating depression has been found to improve adherence [20], cognitive disturbances [62], fatigue [63], quality of life [64], and possibly disease course, by decreasing production of cytokine [18]. There is a growing body of evidence regarding the neuroprotective effects of antidepressants such as fluoxetine [65] and phenelzine [66]. Furthermore, escitalopram has been found to reduce stress-related relapses in a recent open-label, randomized, controlled study [67]. 

Treatment guidelines have been most recently reported by the Canadian Network for Mood and Anxiety Disorders (CANMAT), based on up-to-date literature [68]. Use of antidepressants is strongly recommended, as well as psychotherapies emphasizing coping strategies rather than insight. SSRIs are considered well-tolerated first-line treatment. Drugs with significant sedating or anticholinergic side effects, such as tricyclic antidepressants, should rather be avoided, due to issues with fatigue, orthostatic hypotension, balance, cognitive disturbances, and bladder problems. Patients should also be monitored for manic or hypomanic symptoms, while on antidepressant medication, as the prevalence of bipolar disorder is quite elevated in MS, presumably two to three times higher than in the general population [6].Should depression emerge during treatment with interferon beta, the latter needs not necessarily be discontinued, but the depression should be adequately treated instead [12].

### 3.2. Somatic Treatment

Despite the high burden of depression in multiple sclerosis patients, few trials have been published regarding this particular population, and only two of them were double blind, randomized, placebo controlled, and meeting certain standards of quality, as already observed by Koch et al. [69]. The first one examined the efficacy of desipramine versus placebo and found a trend in favor of desipramine [70]. The second demonstrated greater efficacy of paroxetine versus placebo, with 78.6% versus 42.1% of patients exhibiting response, respectively, but the difference did not reach significance, probably because of underpowering of the study and dosing and duration issues [71]. Another problem faced by both studies was missing data or patients who were lost at followup [69].

In another study by Mohr et al. [72], sertraline was found to be equally effective with cognitive behavioural therapy, and both of them were more efficacious than supportive-expressive therapy in a sample of 63 depressed patients with multiple sclerosis. Open-label studies have demonstrated efficacy of sertraline [73], fluvoxamine [74], moclobemide [75], and fluoxetine [76]. Common side effects were gastrointestinal such as nausea, vomiting, and headache. Unfortunately, evidence documenting therapeutic interventions for the pediatric population is lacking [77].

A few case reports deal with the usefulness of ECT for severe or refractory depression in MS [78]; however, concerns have been raised that ECT might mediate neurological deterioration by altering the number or size of the CNS plaques and/or periplaque edema [79, 80]. It has been postulated that the presence of contrast-enhanced lesions might predict deterioration, and therefore, a gadolinium-enhanced MRI is suggested, but this observation is based on a report of only three patients, one of whom deteriorated after ECT sessions [81].

### 3.3. Psychotherapeutic Interventions

Psychotherapy has long been considered an important treatment option for the management of depression in patients with MS, with approaches focusing on coping skills showing superiority over insight-oriented therapies [82]. Cognitive behaviour therapy (CBT) helps patients to correct distorted cognitive appraisal of the environment and core beliefs that lead to maladaptive behaviour, and change the connection between life events and learned reactions such as depression beliefs [83]. Regarding multiple sclerosis in particular, which imposes progressively increasing physical difficulties and challenges in everyday living, together with cognitive impairment, CBT can help maximize the development of the patients' coping skills [82]. In line with this observation, individual (CBT) has been found more beneficial than supportive expressive therapy (SET), administered either as usual [72] or by telephone [84]. Telephone-administered psychotherapy or counseling is particularly appealing because of the frequent physical disability, which represents an obstacle to receiving therapy otherwise [85]. For similar reasons, computerized forms of CBT are also available [86, 87]. Group CBT has yielded beneficial results, as well [80]. On the other hand, there still may be some patients who would benefit more by insight-oriented, psychodynamic psychotherapy, which focuses on the management of psychological defenses and unconscious psychic activity [82, 88]. The effectiveness of mindful-based intervention (MBI) which is based on the nonjudgmental awareness of everyday moments has also been recently demonstrated [89]. Both CBT and MBI cannot be used by persons with significant cognitive impairment. Finally, other studies have demonstrated the beneficial effects of interpersonal therapy [90]. Individual preference and needs, cognitive performance, and availability of therapist should help the clinician choose among the above diverse psychotherapeutic approaches. 

## 4. Concluding Remarks 

The prevalence of depression in patients is remarkably high, yet it is still frequently underdiagnosed and undertreated. Patients who are not treated are not expected to improve and are at risk for further deterioration. Clinicians should be alert for the risk of suicide and the need for treatment. More and better designed studies for therapeutic interventions, particularly in the pediatric populations, are clearly needed. 
